# Interactions of bile acids and gut microbiota modulate neurological health: a comprehensive review on mechanisms and therapeutic potential of dietary phytochemicals

**DOI:** 10.3389/fmicb.2026.1757551

**Published:** 2026-02-04

**Authors:** Jie Wang, Ying Zhang, Quan Wu, Yingfu Zhong, Ze Xu, Juan Yang

**Affiliations:** Tea Research Institute, Chongqing Academy of Agricultural Sciences, Chongqing, China

**Keywords:** bile acids, gut-brain axis, microbiome, neuroinflammation, neurological health, phytochemicals

## Abstract

Bile acids (BAs), classically regarded as detergents for dietary lipid absorption, have emerged as pivotal signaling molecules with systemic endocrine functions. The discovery of the Farnesoid X Receptor (FXR) and Takeda G protein-coupled receptor 5 (TGR5) as BAs-activated receptors unveiled their profound influences on glucose, lipid, and energy metabolism. BAs are first synthesized in hepatocytes and further metabolized by gut microbes, can either circulate in enterohepatic system or be found in circulations to exert various effects. More recently, the gut-brain axis has been identified as a critical pathway through which BAs exert significant effects on central nervous system (CNS) function and health. Based on research progresses mentioned above, this review systematically delineates the synthesis, metabolism, and classification of BAs, with a focus on the intricate crosstalk between the hepatic-gut BA axis and the brain. In addition, we explore the compelling evidences linking BAs dysregulation to a spectrum of neurological disorders, including neurodegenerative diseases (Alzheimer's and Parkinson's disease), depression, and hepatic encephalopathy. Besides, the potential mechanisms, such as alleviating neuroinflammation, maintaining the integrity of blood-brain barrier, increasing the neuronal survival, and modulating neurotransmitter systems are further elucidated. Finally, strategies of dietary intervention through phytochemicals to modulate the BAs pool for improved neurological outcomes are summarized and discussed. By integrating pre-clinical and clinical findings, this review aims to establish a foundation for understanding BAs as novel therapeutic targets in neurology and nutritional neuroscience.

## 1 Introduction

For centuries, bile has been recognized for its role in digestion. However, the molecular constituents of bile, the bile acids (BAs), have recently been elevated from their humble status as biological detergents to critical signaling molecules with far-reaching systemic effects (Houten et al., 2006). The synthesis of BAs from cholesterol in the liver represents the major pathway for cholesterol catabolism, a process that is not only crucial for lipid homeostasis but also for generating a diverse family of signaling molecules (Chiang, 2013; Liu et al., 2025). Upon meal ingestion, they are released into the duodenum to facilitate the emulsification and absorption of dietary fats and fat-soluble vitamins (Hofmann and Hagey, 2008). The majority (~95%) of BAs are efficiently reabsorbed in the ileum and returned to the liver, a process known as enterohepatic circulation, which ensures their efficient reuse but also creates a dynamic pool that can be influenced by dietary and microbial factors (Dawson and Karpen, 2015). The small fraction that escapes this recycling enters the colon, where the gut microbiota extensively modifies them, generating a diverse secondary BAs pool that possesses distinct biological activities from their primary precursors (Ridlon et al., 2006).

The paradigm shifts in BAs biology began with the identification of specific receptors that recognize BAs as their ligands. The nuclear receptor FXR (Farnesoid X Receptor) and the membrane-bound G protein-coupled receptor TGR5 (Takeda G protein-coupled receptor 5, also known as GPBAR1) are now established as the primary mediators of BA signaling (Makishima et al., 1999; Maruyama et al., 2002). Through these receptors, BAs regulate their own synthesis and enterohepatic circulation, control glucose and lipid homeostasis, modulate energy expenditure, and exert potent anti-inflammatory and metabolic effects across various tissues (Thomas et al., 2008; Pols et al., 2011). The discovery of this “endocrine” dimension of BAs action fundamentally altered our understanding of their physiological roles (Lefebvre et al., 2009).

The influence of BAs extends beyond peripheral tissues to the central nervous system (CNS). The gut-brain axis, a bidirectional communication network linking the gastrointestinal tract and the brain via neural, endocrine, and immune pathways, serves as a critical channel for this crosstalk (Cryan et al., 2019; Sharon et al., 2016). BAs can influence the brain via distinct, though potentially overlapping, pathways: (i) indirect endocrine pathways (e.g., TGR5-mediated GLP-1 secretion, FXR-mediated FGF19 release); (ii) indirect neural pathways (vagal afferent signaling); (iii) modulation of systemic and neuroimmune responses; and (iv) potential direct effects after crossing the BBB (McMillin and DeMorrow, 2016; Grant and DeMorrow, 2020). Dysregulation of BAs homeostasis, often characterized by shifts in the ratio of primary to secondary BAs or in the hydrophobicity profile, has been implicated in a growing list of neurological and psychiatric conditions, suggesting a core disruption of gut-brain communication (Kiriyama and Nochi, 2019; Jia et al., 2018). Conversely, neurological states, particularly those involving stress, can influence gut permeability and motility, thereby shaping the BA metabolism, creating a complex, dynamic feedback loop (Foster et al., 2017; Collins et al., 2023).

This review aims to synthesize the current knowledge on the classification, synthesis, and metabolism of BAs, and to critically evaluate their emerging role as key modulators of brain health and disease. To identify relevant literature, we performed searches in PubMed, Web of Science, and Google Scholar databases from inception until October 2025, using keywords and their combinations including: “bile acid,” “FXR,” “TGR5,” “gut-brain axis,” “neurodegeneration,” “depression,” “hepatic encephalopathy,” “microbiome,” and “phytochemical.” Given the interdisciplinary and emerging nature of the topic, we prioritized inclusion of high-impact original research, authoritative reviews, and seminal clinical studies. We will delve into the molecular mechanisms connecting specific BA alterations to the pathogenesis of neurodevelopmental, neurodegenerative, and neuropsychiatric disorders. Finally, we will conclude by exploring the exciting potential of targeted dietary interventions, particularly through the use of plant-derived phytochemicals, as a strategic means to modulate BAs for the prevention and adjunct treatment of neurological conditions, thereby opening a new frontier in nutritional neuroscience.

## 2 Classification and sources of BAs

**Figure 1:**
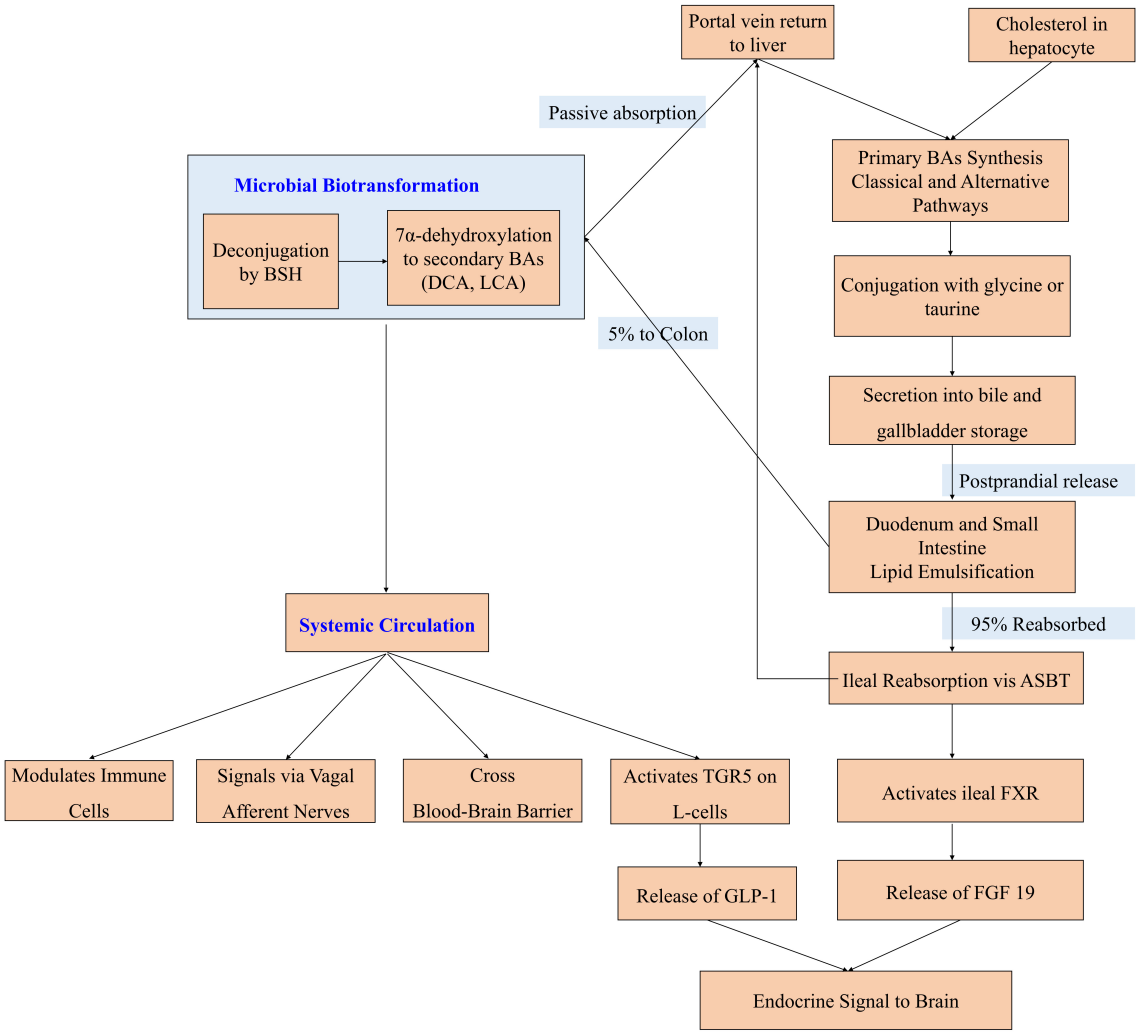
The enterohepatic circulation and gut-brain axis of BAs. The enterohepatic circulation include: (1) Hepatocyte: primary BAs (Cholic Acid, CA; Chenodeoxycholic Acid, CDCA) are synthesized from cholesterol via the classical pathway (CYP7A1) and the alternative pathway (CYP27A1/CYP7B1). (2) Conjugation: primary BAs are conjugated with glycine or taurine (via BAAT) to form conjugated BAs (e.g., TCA, GCA). (3) Gallbladder & secretion: conjugated BAs are stored in the gallbladder and released into the duodenum upon meal ingestion. (4) Emulsification: BAs facilitate the emulsification and absorption of dietary lipids and fat-soluble vitamins in the small intestine. (5) Ileal reabsorption: ~95% of BAs are actively reabsorbed in the ileum via the Apical Sodium-dependent Bile Acid Transporter (ASBT) and returned to the liver via the portal vein. (6) Colonic biotransformation: the remaining BAs reach the colon, where gut microbiota performs deconjugation (Bile Salt Hydrolase, BSH) and 7α-dehydroxylation to form secondary BAs (Deoxycholic acid, DCA; Lithocholic acid, LCA). A portion of these are passively absorbed and returned to the liver. The gut-brain signaling pathways include: (1) Systemic circulation & BBB: BAs can enter systemic circulation and some may cross the blood-brain barrier to act directly on neurons and glial cells (e.g., via FXR, TGR5). (2) Neural & immune pathways: BAs can signal via the vagus nerve or by modulating the activity of systemic and perivascular immune cells. (3) Endocrine pathways: ileal FXR Activation by BAs induces the release of Fibroblast Growth Factor 19 (FGF19), which can signal to the brain. TGR5 Activation on intestinal L-cells stimulates the secretion of Glucagon-Like Peptide-1 (GLP-1), which has central effects.

The BAs pool in humans is a complex mixture of different steroid molecules, primarily classified based on their origin and structure. This diversity is fundamental to their varied signaling functions and their impact on host physiology, including neurology (Chiang, 2009). The classification and properties of major human BAs are shown in Table 1. The enterohepatic circulation and gut-brain axis of BAs are presented in Figure 1.

**Table 1:** Major human bile acids: classification and properties.

**Bile acid**	**Abbreviation**	**Type**	**Origin**	**Hydrophobicity index**	**Primary receptor affinity**
Cholic acid	CA	Primary	Hepatic synthesis	0.00	FXR (weak)
Chenodeoxycholic acid	CDCA	Primary	Hepatic synthesis	0.59	FXR (potent)
Deoxycholic acid	DCA	Secondary	Bacterial 7α-dehydroxylation of CA	0.79	FXR, TGR5
Lithocholic acid	LCA	Secondary	Bacterial 7α-dehydroxylation of CDCA	0.96	PXR, VDR, TGR5
Ursodeoxycholic acid	UDCA	Secondary	Bacterial epimerization of CDCA	0.08	Antagonizes FXR, TGR5
Glycocholic acid	GCA	Primary (conjugated)	Conjugation of CA with glycine	−0.13	TGR5
Taurochenodeoxycholic acid	TCDCA	Primary (conjugated)	Conjugation of CDCA with taurine	−0.22	TGR5?

FXR: Farnesoid X Receptor; TGR5: Takeda G protein-coupled receptor 5; PXR: Pregnane X Receptor; VDR: Vitamin D Receptor.

### 2.1 Primary and secondary bile acids

Primary BAs are synthesized *de novo* from cholesterol in the hepatocytes of the liver. The two main primary BAs in humans are cholic acid (CA) and chenodeoxycholic acid (CDCA; Sayin et al., 2013). A critical interspecies difference exists; mice and rats produce significant amounts of muricholic acids (MCAs), particularly β-muricholic acid (β-MCA), which acts as a natural FXR antagonist. This distinction is crucial when extrapolating findings from animal models to human physiology and therapeutic development (Li-Hawkins et al., 2002). Before secretion into bile, primary BAs are almost always conjugated at the C-24 carboxyl group with either the amino acids glycine or taurine, forming conjugated BAs such as glycocholic acid (GCA), taurocholic acid (TCA), glycochenodeoxycholic acid (GCDCA), and taurochenodeoxycholic acid (TCDCA; Hofmann, 1999). Conjugation, catalyzed by the enzyme bile acid-CoA: amino acid N-acyltransferase (BAAT), can increase the hydrophilicity and acidity of BAs, and therefore reduce their passive absorption in the biliary tract and small intestine. This ensures their efficient delivery to the distal gut where they are needed for both lipid digestion and microbial transformation, thereby linking hepatic synthesis with gut microbial ecology (Dawson et al., 2009; Stieger, 2010).

Secondary BAs are derived from primary BAs through the enzymatic activities of the gut microbiota in the colon, a process known as biotransformation that significantly expands the functional repertoire of the BAs pool (Wahlström et al., 2016). This process involves two key steps (Ridlon et al., 2016a). The first step is deconjugation in which bacterial bile salt hydrolases (BSH) cleave the glycine/taurine conjugate, releasing free, unconjugated primary BAs (CA and CDCA). BSH enzymes are widely distributed across many bacterial phyla, including *Firmicutes* (e.g., *Lactobacillus, Clostridium*), *Bacteroidetes*, and *Actinobacteria* (e.g., *Bifidobacterium*), making this a common microbial function (Jones et al., 2008; Foley et al., 2019). The second step is 7α-dehydroxylation. This is a more specialized subset of anaerobic bacteria, notably from the genera *Clostridium* (e.g., *C. scindens*) and *Eubacterium*, performs 7α-dehydroxylation. This complex enzymatic process removes the 7α-hydroxyl group, converting CA into deoxycholic acid (DCA) and CDCA into lithocholic acid (LCA; Little et al., 2020; Winston and Theriot, 2020). LCA is highly hydrophobic and relatively insoluble with a significant portion excreted into feces, while some is absorbed and sulfated in the liver for renal excretion (Stiehl et al., 1980). DCA is more efficiently absorbed and becomes a major component of the circulating BA pool, contributing significantly to BA signaling (Hamilton et al., 2007). Other bacterial modifications include oxidation and epimerization of hydroxyl groups, leading to the formation of minor but physiologically important BAs like ursodeoxycholic acid (UDCA; the 7β-epimer of CDCA) and various iso-BAs, which can have unique receptor affinities (Batta et al., 1990; Ridlon et al., 2014).

A third category, sometimes termed tertiary BAs, includes BAs that undergo further hepatic modification after intestinal absorption, such as the re-conjugation of secondary BAs (e.g., glycodeoxycholic acid, GDCA) or the sulfation of LCA to increase its solubility for renal excretion, representing the liver's role in “editing” the microbiome-modified BA pool (Zollner and Trauner, 2008; Jansen et al., 2017).

### 2.2 Synthesis of primary bile acids

The *de novo* synthesis of primary BAs from cholesterol is a complex, multi-step process that occurs in the hepatocyte and is tightly regulated to maintain BA homeostasis and overall cholesterol balance. It can be divided into two major pathways that operate in parallel (Chiang, 2002).

1. **The classical (neutral) pathway:** This is the predominant pathway in humans, responsible for about 75% of the total BAs synthesis. It is initiated by the rate-limiting enzyme cholesterol 7α-hydroxylase (CYP7A1), which hydroxylates cholesterol at the 7α position (Russell and Setchell, 1992). The subsequent activity of sterol 12α-hydroxylase (CYP8B1) is a key branch point, determining the ratio of CA to CDCA produced. The high CYP8B1 activity is beneficial for producing CA with stronger hydrophilicity, while low activity shifts production toward CDCA (Pandak and Kakiyama, 2019).
2. **The alternative (acidic) pathway:** This pathway contributes to the remaining 25% of BA production and is initiated by mitochondrial sterol 27-hydroxylase (CYP27A1), followed by oxidation by oxysterol 7α-hydroxylase (CYP7B1). This pathway primarily leads to the formation of CDCA and becomes relatively more important when the classical pathway is impaired (Li and Chiang, 2012).

The synthesis of BAs is under exquisite negative feedback regulation, primarily mediated by the FXR-SHP (Small Heterodimer Partner) axis. When intrahepatic BA levels rise, particularly of potent agonists like CDCA, they activate FXR (Lu et al., 2000). Activated FXR induces the expression of SHP, which in turn represses the transcription of the genes encoding *CYP7A1* and *CYP8B1*, thus reducing the amount and altering the composition of BA produced (Goodwin et al., 2000; Wang et al., 2002). An additional endocrine loop involves the ileum: upon BA absorption, intestinal FXR is activated, leading to the secretion of fibroblast growth factor 19 (FGF19; FGF15 in mice) into the portal circulation (Inagaki et al., 2005). FGF19 travels to the liver and binds to its receptor FGFR4/β-Klotho complex, initiating a signaling cascade that potently represses *CYP7A1* transcription, providing a refined, meal-responsive layer of regulation (Potthoff et al., 2013; Song and Chiang, 2006).

### 2.3 Synthesis of secondary bile acids

The synthesis of secondary BAs is entirely dependent on the metabolic activity of the gut microbiome, making it a key point of interaction between host physiology and microbial ecology (Theriot and Young, 2015). As conjugated primary BAs traverse the small intestine and reach the colon, they encounter a dense and diverse microbial community equipped with a repertoire of BA-transforming enzymes (Devkota et al., 2012). The key enzymes involved are bile salt hydrolases (BSH) and 7α-dehydroxylase (Foley et al., 2019).

BSH present in many commensal bacteria (e.g., *Lactobacillus, Bifidobacterium, Clostridium, Bacteroides*), are the gatekeepers of secondary BAs formation (Joyce and Gahan, 2014). Deconjugation is a prerequisite for most subsequent bacterial modifications, particularly 7α-dehydroxylation. BSH activity is thought to provide a fitness advantage to bacteria by reducing BA toxicity in the gut environment and by providing a source of glycine and taurine, which can be used as nutrients or for sulfur metabolism (Foley et al., 2019).

7α-Dehydroxylase is a multi-enzyme complex found in a more restricted, specialized group of Gram-positive anaerobes (e.g., *Clostridium scindens*). It catalyzes the removal of the 7α-hydroxyl group, converting CA to DCA and CDCA to LCA (Dean et al., 2020). This pathway is highly sensitive to the colonic environment, particularly pH. A lower pH resulting from bacterial fermentation of dietary fiber to short-chain fatty acids can inhibit 7α-dehydroxylase activity, thereby providing a direct link between diet, microbial metabolism, and the secondary BAs profile (Islam et al., 2011; Ridlon et al., 2016b).

The composition and metabolic capacity of the gut microbiome therefore directly shapes the secondary BAs metabolome. Factors such as diet (high-fat, high-fiber), antibiotics, age, and various disease states can dramatically alter the microbial community structure, particularly the abundance of 7α-dehydroxylating bacteria, and consequently, the profile of secondary BAs (Jiao et al., 2018; Mouzaki et al., 2016). This has profound implications for systemic health, including immune function, metabolic homeostasis, and, as discussed in subsequent sections, brain function and disease susceptibility (Schaap et al., 2014; de Aguiar Vallim et al., 2013).

## 3 The crosstalk between metabolism of BAs and gut microbiota

### 3.1 BAs affect the gut microbiota and its composition

Various BAs in the intestine can directly or indirectly inhibit the growth of gut microbiota especially when primary BAs (CA) is converted into secondary BAs (DCA) through dehydroxylation, its antibacterial ability can be increased by more than 10 times. Low doses of various BAs can affect the fluidity and permeability of bacterial cell membranes. In contrast, high doses can directly bind to membrane phospholipids and disrupt internal protein structures. This disruption causes cellular enzymes and contents to leak out, thereby inhibiting excessive intestinal microbiota growth (Malhi and Camilleri, 2017). The increase in BAs secretion and changes in BAs composition caused by high-fat diet can significantly affect the types and quantities of gut microbiota, and different gut microbiota members have varying sensitivities to different BAs components (Janssen et al., 2017).

Feeding BAs to rats can increase the abundance of *Firmicutes* and decrease the abundance of *Bacteroidetes*, ultimately leading to an increase in the ratio between *Firmicutes* and *Bacteroidetes*. Feeding milk derived fat to mice leads to an increased level of TCA. And the abundance of opportunistic pathogens such as *Bilophila wadsworth* is also increased (Schneider et al., 2018). Janssen et al. (2017) found that feeding mice with TCA not only causes changes in gut microbiota structure, but also exacerbates liver inflammation and fibrosis, indicating that BAs metabolism can indirectly affect gut microbiota structure by inducing immune responses in the liver to inhibit the growth of certain specific microorganisms. After binding to FXR, BAs can regulate the gene expression of antibacterial substances in the intestine, and prevent the overgrowth of bacterial by activating the defense system of the small intestine (Buzzetti et al., 2016). After binding to FXR, CDCA can cause intestinal epithelial cells to secrete antimicrobial peptides with broad-spectrum antibacterial activity (such as cathelicidin), further exerting antibacterial effects (Rodrigues et al., 2014). In addition, a high-fat diet causes the accumulation of liver cell fat and insulin resistance. These changes lead to BAs accumulation and compositional shifts, which in turn cause an imbalance in the gut microbiota. This microbiota imbalance further exacerbates BAs metabolism disorders and liver inflammatory reactions (Inagaki et al., 2006).

### 3.2 Gut microbiota influences and participates in the metabolism of BAs

The synthesis of BAs in the liver begins with a cytochrome P450-mediated oxidation of cholesterol. This reaction primarily generates CA and CDCA through classical and alternative pathways. These acids then conjugate with taurine or glycine to form conjugated BAs (Wahlström et al., 2016). The synthesized primary BAs are stored in the gallbladder through a bile salt export pump, and the contraction of the gallbladder caused by eating can promote the secretion of BAs into the intestine. The BSH enzymes produced by *Bacteroides, Clostridium, Lactobacillus, Bifidobacterium*, and *Listeria* in the gut microbiota can cause the dissociation of bound BAs to form free BAs. *Clostridium, Fusobacterium, Peptococcus*, and *Pseudomonas* can catalyze the desulfurization of BAs. *Bacteroides, Eubacteria, Clostridium, Escherichia, Eggerthella, Peptostreptococcus*, and *Ruminococcus* can remove hydroxyl groups on C3, C7, and C12 to form secondary BAs such as DCA and LCA (He et al., 2016). About 95% of primary and secondary BAs can be reabsorbed in the intestine and transported back to the liver through the portal vein, but LCAin secondary BAs is mostly excreted with feces. The BAs lost in the enterohepatic circulation are replenished by the synthesis of new BAs by liver. After BAs reabsorption, it binds to FXR and TGR-5 in ileal, promoting the secretion of FGF-19 by intestinal. The latter then binds to FGFR-4 and activates the c-Jun N-terminal kinase and extracellular signal regulated kinase signaling pathways, respectively, to reduce the gene expression of *CYP7A1* enzyme. Thus, it inhibits the synthesis of BAs in a negatively feedback way (Chiang, 2017).

## 4 BAs and brain disorders

The connection between BAs metabolism and neurological health is supported by a rapidly expanding body of evidences from metabolomic studies, animal models, and clinical observations. Alterations in the BAs pool, either in overall composition, concentration, or the relative abundance of specific species, can disrupt central homeostatic mechanisms and contribute to disease pathogenesis through multiple interconnected pathways.

### 4.1 BAs and neurodegenerative diseases

#### 4.1.1 Alzheimer's disease (AD)

AD is characterized by the accumulation of amyloid-β (Aβ) plaques and neurofibrillary tangles of hyperphosphorylated tau, accompanied by chronic neuroinflammation and synaptic loss. Recent metabolomic studies have consistently identified significantly altered BAs profiles in the serum, plasma, and cerebrospinal fluid of AD patients and those with mild cognitive impairment (Baloni et al., 2020; MahmoudianDehkordi et al., 2019). A common finding is a relative increase in conjugated primary BAs (e.g., TCA, GCA) and a decrease in secondary BAs, particularly DCA and its conjugates (Nho et al., 2019; Marksteiner et al., 2018). This pattern suggests a potential shift toward reduced microbial 7α-dehydroxylation activity, possibly reflecting the gut dysbiosis reported in AD patients. While causal relationships remain to be firmly established, several mechanistic pathways, primarily elucidated in preclinical models, provide plausible links between these BA alterations and AD pathophysiology TGR5 Signaling in Neuroinflammation: The TGR5 receptor is expressed on key CNS cell types, including microglia, the brain's resident immune cells (Keitel et al., 2010). Preclinical studies indicate that activation of TGR5 by BA ligands like TUDCA, LCA, or DCA has been shown to induce a potent anti-inflammatory response. It shifts microglia from a pro-inflammatory (M1) state, characterized by the production of neurotoxic cytokines like TNF-α, IL-1β, and IL-6, to an anti-inflammatory (M2) phenotype that promotes tissue repair and resolution of inflammation (Yoneno et al., 2013; Guo et al., 2016). This TGR5-mediated suppression of neuroinflammation is hypothesized to be a key mechanism through which BAs could potentially mitigate AD progression. Furthermore, TGR5 activation in astrocytes and neurons promotes mitochondrial function and reduces oxidative stress, both of which are compromised in AD (McMillin et al., 2015; Keitel and Häussinger, 2011).

FXR Signaling in Amyloid and Tau Pathology: FXR is also expressed in the brain, with notable presence in neurons (Huang et al., 2016). FXR activation has been demonstrated to influence amyloid precursor protein (APP) processing, potentially reducing the production of pathogenic Aβ peptides by downregulating the expression and activity of β-secretase (BACE1; Mulak, 2021). Furthermore, FXR agonism has been shown to improve cognitive function and reduce Aβ plaque load in transgenic AD mouse models. Emerging evidence also suggests that FXR signaling can modulate tau phosphorylation, indicating a broader role in attenuating key aspects of AD pathology (Ackerman and Gerhard, 2016).

Direct Neuroprotection by TUDCA: The taurine-conjugated BA Tauroursodeoxycholic Acid (TUDCA), and its unconjugated form UDCA, have demonstrated particularly potent neuroprotective properties in various models of neurodegeneration (Vang et al., 2014). TUDCA acts as a chemical chaperone that reduces Endoplasmic Reticulum (ER) stress, a key contributor to neuronal apoptosis in AD (Ramalho et al., 2008). It also stabilizes the mitochondrial membrane by inhibiting the mitochondrial Permeability Transition Pore (mPTP), thereby preventing the release of pro-apoptotic factors like cytochrome c, and attenuates apoptosis by modulating the balance of Bcl-2 family proteins (Rodrigues et al., 2002). In preclinical studies, administration of TUDCA reduces both Aβ and tau pathology, mitigates synaptic loss, and improves memory and learning performance (Nunes et al., 2012).

Blood-Brain Barrier Integrity: The BBB is increasingly recognized as a critical player in AD pathogenesis. BAs can exert dual effects on the BBB. High concentrations of hydrophobic BAs like CDCA and DCA can be cytotoxic and disrupt tight junction proteins (e.g., occludin, ZO-1), increasing BBB permeability. In contrast, UDCA and TUDCA have been shown to protect BBB integrity by reducing inflammation and oxidative stress in brain endothelial cells, thereby limiting the entry of neurotoxic blood-derived substances into the brain parenchyma (Yanguas-Casás et al., 2017).

#### 4.1.2 Parkinson's disease (PD)

PD is marked by the progressive loss of dopaminergic neurons in the substantia nigra pars compacta and the presence of intracellular Lewy bodies composed of aggregated α-synuclein. The gut-brain axis is strongly implicated in PD, with Braak's hypothesis proposing that α-synuclein pathology may originate in the enteric nervous system and spread to the CNS via the vagus nerve (Sampson et al., 2016). BAs alterations increasingly recognized as part of this gut-brain dialogue in PD in several ways are discussed below.

The first one is altered BAs metabolism and dysbiosis. PD patients frequently show altered BA profiles in their feces and serum, often characterized by decreased levels of secondary BAs, particularly DCA and LCA (Hasegawa et al., 2015; Li et al., 2021). This is consistent with the well-documented gut dysbiosis in PD, which often includes a reduced abundance of bacterial genera from the *Clostridium* clusters that are capable of 7α-dehydroxylation (e.g., *Clostridium scindens*; Scheperjans et al., 2015; Hill-Burns et al., 2017). The reduction in DCA is particularly significant as it is a potent TGR5 agonist, and its deficiency could diminish a key anti-inflammatory signaling pathway in the brain and periphery.

The second one is FXR and TGR5 in glial neuroprotection. Similar to AD, the anti-inflammatory effects of FXR and TGR5 signaling are highly relevant to PD, where neuroinflammation driven by activated microglia contributes to dopaminergic neuron loss (Wang et al., 2008). Activation of these receptors on glial cells can suppress the production of pro-inflammatory cytokines and reactive oxygen species, creating a more supportive neuronal environment (Mencarelli et al., 2009). Additionally, TGR5 activation in intestinal L-cells promotes the release of GLP-1, and GLP-1 receptor agonists have shown promising neuroprotective and anti-inflammatory effects in PD models, independently improving motor function and neuronal survival (Hölscher, 2018; Athauda and Foltynie, 2016).

Besides, modulation of α-synuclein aggregation may also contribute to the progression of PD. While the direct interaction between BAs and α-synuclein is still an emerging area of research, it is hypothesized that the gut microbial environment and its BAs metabolites could influence α-synuclein aggregation in the enteric nervous system or its propagation to the brain (Devos et al., 2013). The systemic anti-inflammatory environment fostered by certain BAs might reduce the glial activation that facilitates the spread and toxicity of α-synuclein pathology (Fiorucci et al., 2018). Furthermore, the ability of BAs like UDCA to act as chemical chaperones could theoretically mitigate α-synuclein misfolding, and reducing oxidative stress and neuroinflammation through mitogen-activated protein kinases pathways in MPTP-induced mouse model of PD (Keene et al., 2001; Jiang et al., 2022).

### 4.2 BAs and depression

Major Depressive Disorder (MDD) is a multifactorial disease characterized by complex interactions between genetic predisposition, chronic stress, neuroinflammation, hypothalamic-pituitary-adrenal (HPA) axis dysregulation, and monoaminergic neurotransmitter dysfunction. The BAs system intersects with these core pathophysiological pathways in several compelling ways summarized below.

**HPA Axis Modulation:** The HPA axis, the body's central stress response system, is often hyperactive in depression. BAs can cross the BBB and interact with receptors in key regulatory regions like the hypothalamus and pituitary (Huang et al., 2015). Certain BAs, notably LCA, are potent agonists of the Pregnane X Receptor (PXR) and Vitamin D Receptor (VDR). Both of them can cross-talk with glucocorticoid receptor signaling and influence HPA axis feedback sensitivity (Staudinger et al., 2001; Makishima et al., 2002). An imbalanced BAs profile, particularly one enriched in specific secondary BAs, may therefore contribute to the HPA axis hyperactivity seen in depression. Preclinical evidence supports this; for instance, FXR knockout mice exhibit exacerbated depressive-like behaviors and impaired HPA axis negative feedback in response to chronic stress, suggesting the role of FXR in stress resilience (Hu et al., 2020; Huang et al., 2015).

**Neurotransmitter Systems:** BAs can directly and indirectly modulate the activity of central neurotransmitter systems. Some BAs have been shown to act as allosteric modulators of neurotransmitter receptors, including the gamma-aminobutyric acid-A (GABA-A) receptor. For example, certain BAs can potentiate GABAergic inhibition, which may influence neural circuit activity underlying anxiety and mood (Quinn et al., 2014; Ahboucha and Butterworth, 2008). More indirectly, the TGR5-mediated release of GLP-1 from intestinal L-cells can affect central dopamine and serotonin signaling pathways, both of which are critically implicated in the pathophysiology and treatment of depression (Ghosal et al., 2013; Harach et al., 2012).

**Inflammation and Neurogenesis:** Depression is now recognized as a neuroinflammatory disorder, with elevated levels of pro-inflammatory cytokines contributing to symptoms and negatively impacting neurogenesis, particularly in the hippocampus (Miller and Raison, 2016). The anti-inflammatory effects of TGR5 and FXR signaling, as described in neurodegenerative contexts, are therefore highly relevant. By reducing the production of pro-inflammatory cytokines (e.g., IL-6, TNF-α) in both the periphery and the brain, a healthy, balanced BAs profile may help to create an environment conducive to hippocampal neurogenesis, which is often impaired in depression and is thought to be a mechanism of action for some antidepressants (Lucidi et al., 2021; Miller and Raison, 2016). Administration of TUDCA has been shown to reduce depressive-like behaviors in rodent models, an effect associated with mitigated neuroinflammation, reduced oxidative stress, and promoted synaptic plasticity and neurogenesis (Ackerman and Gerhard, 2016; Yanguas-Casás et al., 2017).

**Clinical and Metabolomic Evidences:** Metabolomic analyses in human patients consistently report that altered serum and plasma BAs profiles in individuals with MDD compared to healthy controls. While findings can vary, a recurring result is a disturbance in the ratio of primary to secondary BAs. Research has revealed a specific shift in the BAs profile: an increase in primary conjugated forms alongside a decrease in secondary ones like DCA and LCA. This shift mirrors a “dysbiotic” signature seen in neurodegenerative diseases. Furthermore, it strengthens the evidence for a shared gut-brain axis disruption in various neuropsychiatric conditions (Zheng et al., 2016). A larger-scale study found that specific BAs ratios and concentrations were correlated with depression severity. Importantly, these alterations were partially normalized after successful antidepressant treatment. This suggests the changes are state-dependent and hold potential for monitoring treatment response (Jia et al., 2024).

### 4.3 BAs and hepatic encephalopathy

Hepatic Encephalopathy (HE) is a complex and debilitating neuropsychiatric complication of acute or chronic liver failure, characterized by a wide spectrum of symptoms from subtle cognitive impairment to coma. While hyperammonemia is a central driver, the role of BAs in HE pathogenesis is gaining significant attention, as they are intimately linked to liver function and can directly impact brain function (Ahluwalia et al., 2016). The neurotoxic mechanism of BAs in HE involves multiple pathways.

**The Accumulation and Altered Composition of Peripheral BAs:** In advanced liver cirrhosis and liver failure, the damaged liver fails to synthesize, conjugate, and clear BAs efficiently. This leads to systemic BAs accumulation, a condition known as cholestasis (Jansen et al., 2017). The composition of the circulating BA pool becomes profoundly altered, typically featuring an increase in the concentrations of toxic, hydrophobic BAs like CDCA and DCA, and a relative decrease in the more hydrophilic and less toxic BAs like CA and UDCA (Kakiyama et al., 2013; Ferslew et al., 2015). This shift toward a more hydrophobic and cytotoxic BAs profile is a major contributor to systemic and cerebral toxicity in HE.

**Disruption of BBB:** The BBB is a critical interface that becomes compromised in HE. High concentrations of hydrophobic BAs are directly cytotoxic to cerebrovascular endothelial cells. They can disrupt the integrity of tight junctions (e.g., by downregulating occludin and ZO-1 expression and promoting oxidative stress), leading to increased BBB permeability (Görg et al., 2015). This “leaky” BBB facilitates the entry of neurotoxins such as ammonia, manganese, and pro-inflammatory cytokines into the brain parenchyma, where they can disrupt astrocyte function (leading to Alzheimer type II astrocytosis) and neuronal excitability (Ziemińska and Lazarewicz, 2006; Ahboucha and Butterworth, 2008).

**Direct Neurotransmitter Dysfunction:** BAs can directly interfere with neuronal and synaptic function. They have been shown to alter neuronal membrane fluidity and inhibit key enzymes like Na+/K+-ATPase, which is essential for maintaining the neuronal resting membrane potential and ionic gradients (Ziemińska and Lazarewicz, 2006). This inhibition can contribute to the overall neural inhibition and cognitive-motor slowing characteristic of HE. Furthermore, some BAs and their conjugates can potentiate GABAergic neurotransmission by acting on the GABA-A receptor, potentially exacerbating the sedative effects and neural inhibition observed in HE patients (Ahboucha and Butterworth, 2008; Quinn et al., 2014).

**Therapeutic Applications of UDCA and TUDCA:** Given their well-established cytoprotective, anti-apoptotic, and anti-inflammatory properties, UDCA and its taurine conjugate TUDCA are approved for the treatment of certain cholestatic liver diseases like primary biliary cholangitis (Paumgartner and Beuers, 2002; Rodrigues and Steer, 2001). Their mechanism of action in this context is thought to involve, in part, the displacement of toxic endogenous BAs from the circulating pool. In the context of HE, by stabilizing hepatocyte and neuronal membranes, reducing oxidative stress, improving mitochondrial function, and protecting BBB integrity, UDCA/TUDCA have the potential to ameliorate HE symptoms (Beuers et al., 2015; Halilbasic et al., 2015). Studies in animal models of acute liver failure and bile duct ligation have shown that TUDCA treatment can significantly reduce brain edema, lower BBB permeability, attenuate neuroinflammation, and improve neurological outcomes (Yanguas-Casás et al., 2017; Rodrigues et al., 2003).

### 4.4 BAs and other neurological conditions

Observational and preclinical studies are exploring the potential neuromodulatory and neuroprotective roles of BAs in a wider spectrum of CNS disorders, revealing a common thread of gut-brain communication.

#### 4.4.1 Multiple sclerosis (MS)

As a prototypical autoimmune, neuroinflammatory disease driven by T-cell mediated attack on CNS myelin, MS is strongly influenced by immunomodulatory pathways that BAs can regulate. TGR5 activation on monocytes, macrophages, and microglia can suppress the differentiation and effector functions of pro-inflammatory Th1 and Th17 cells, which are key drivers of MS pathology (Wang et al., 2011; Fiorucci et al., 2018). Studies in the experimental autoimmune encephalomyelitis (EAE) mouse model, a classic model of MS, have demonstrated that administration of FXR or TGR5 agonists can ameliorate disease severity, reduce CNS infiltration of inflammatory cells, decrease demyelination, and promote an anti-inflammatory microenvironment (Vidicevic et al., 2024). Notably, a seminal study found that MS patients had altered serum BA profiles, and that supplementation with taurine-conjugated Ursodeoxycholic Acid (TUDCA) specifically ameliorated neuroinflammation and clinical scores in the EAE model, highlighting a direct therapeutic link (Bhargava et al., 2020).

#### 4.4.2 Ischemic and hemorrhagic stroke

The robust neuroprotective properties of TUDCA have been extensively documented in models of cerebral ischemia. In both transient and permanent middle cerebral artery occlusion models, TUDCA administration reduces infarct volume, attenuates neuronal apoptosis, decreases BBB disruption, and promotes long-term functional recovery (Rodrigues et al., 2003; Viana et al., 2009). The underlying mechanisms are multifactorial, involving the stabilization of mitochondria via inhibition of the mPTP, reduction of ER stress through its chaperone activity, decreased activation of caspases, and modulation of anti-apoptotic Bcl-2 proteins (Rodrigues et al., 2002; Vang et al., 2014). These findings suggest BAs-based therapies as promising candidates for acute neuroprotection in stroke.

#### 4.4.3 Amyotrophic lateral sclerosis (ALS)

ALS is a devastating neurodegenerative disease characterized by the progressive loss of upper and lower motor neurons. TUDCA has shown significant promise in preclinical models of ALS. In the SOD1-G93A transgenic mouse model, TUDCA treatment delayed disease onset, extended survival, reduced motor neuron loss, and decreased markers of ER stress and apoptosis in the spinal cord (Elia et al., 2016; Vaz et al., 2015). This compelling preclinical data propelled TUDCA into clinical trials. A randomized, placebo-controlled trial in ALS patients demonstrated that TUDCA was safe and well-tolerated and showed a encouraging trend toward slowing functional decline, leading to its inclusion in larger, multi-center Phase 3 clinical trials to definitively assess its efficacy (Paganoni et al., 2020).

#### 4.4.4 Huntington's disease (HD)

HD is an inherited neurodegenerative disorder caused by a CAG repeat expansion in the huntingtin gene, leading to protein misfolding and selective neuronal loss in the striatum. In models of HD, such as the 3-nitropropionic acid toxin model and genetic models, TUDCA treatment has been shown to improve motor performance, reduce striatal neuron degeneration, and decrease markers of mitochondrial dysfunction and caspase activation (Keene et al., 2001; Rodrigues and Steer, 2001). This suggests that the anti-apoptotic and protein-stabilizing properties of BAs like TUDCA may have broad application across a range of protein-misfolding and neurodegenerative disorders. The study conducted by Lombardo et al., despite being carried out during the COVID-19 pandemic, did not reach the primary endpoint; however, TUDCA was associated with an excellent safety profile (Lombardo et al., 2023).

## 5 Potential dietary intervention strategies based on BAs regulation

Given the profound impact of diet on both the gut microbiota composition and function, and on host BAs metabolism, dietary intervention represents a promising, accessible, and non-pharmacological approach to modulate the BAs pool in benefiting neurological health. The overarching goal is to shift the BAs pool away from a “dysbiotic” profile and toward a “healthy” one. The dysbiotic profile is characterized by high levels of hydrophobic, cytotoxic BAs (like CDCA and LCA) and a low abundance of beneficial secondary BAs. In contrast, the healthy profile is more balanced, hydrophilic, and enriched with neuroprotective species like UDCA/TUDCA (Wahlström et al., 2016). Phytochemicals—bioactive compounds derived from plants—are particularly potent and versatile modulators of this system. Selected dietary components and their proposed mechanisms of BAs modulation and neurological impact are shown in Table 2.

**Table 2:** Selected dietary components and their proposed mechanisms of bile acid modulation and neurological impact.

**Dietary component**	**Example sources**	**Proposed mechanism on BA system**	**Potential neurological benefit**
Resveratrol	Grapes, red wine, berries	FXR activation; modulates gut microbiota composition and function	Neuroprotection in AD/PD models; reduces neuroinflammation and oxidative stress
Curcumin	Turmeric	Weak FXR agonist; TGR5 modulation; Profoundly shapes microbiome	Anti-depressant and anxiolytic effects; ameliorates AD pathology and cognitive decline
Berberine	Goldenseal, barberry	Inhibits bacterial BSH; alters BA pool composition; Enhances intestinal FXR/FGF19 signaling	Anti-depressant; Improves cognition in AD models; metabolic benefits that support brain health
Quercetin	Onions, apples, capers	TGR5 activation; modulates gut microbiota	Neuroprotection in stroke and AD models; anti-inflammatory and antioxidant
Soluble fiber	Oats, legumes, apples	Promotes SCFA production; lowers colonic pH and inhibits 7α-dehydroxylating bacteria	Reduces systemic inflammation; supports gut-brain axis health; lowers risk of cognitive decline
Resistant starch	Cooked & cooled potatoes, green bananas	Promotes growth of non-7α-dehydroxylating bacteria (e.g., *Bifidobacterium*)	Improves metabolic health and insulin sensitivity, indirectly benefiting the brain
Taurine	Seafood, meat	Substrate for taurine conjugation of BAs, promoting formation of forms like TUDCA	Supports endogenous neuroprotective BA production; direct neuroprotective effects

### 5.1 Polyphenols and alkaloids

**Resveratrol (found in grapes, berries, and peanuts):** This well-studied compound has been shown to act as an FXR agonist, inducing the expression of SHP and thereby contributing to the feedback repression of hepatic BA synthesis (Qiao et al., 2014). Perhaps more importantly, resveratrol significantly modulates the gut microbiota by promoting beneficial bacteria like Lactobacillus and Bifidobacterium. It may also suppress pathobionts, and these combined effects can indirectly influence the production of secondary Bas (Larrosa et al., 2009). It has been well-documented that resveratrol has neuroprotective effects in AD and PD models. These effects include reducing Aβ plaques, attenuating neuroinflammation, and improving cognitive and motor function. These benefits may be partially achieved through the BAs-FXR axis and its subsequent anti-inflammatory and metabolic actions (Bastianetto et al., 2015; Tellone et al., 2015).

**Curcumin (the primary curcuminoid in turmeric):** Curcumin exhibits a complex relationship with BAs signaling. It is a weak FXR agonist and can also modulate TGR5 signaling, potentially contributing to its metabolic benefits (Yang et al., 2016). Its most significant impact may be on the gut microbiome. Curcumin consistently alters microbial composition, often promoting the growth of bacteria associated with a healthy BAs metabolism (e.g., *Bifidobacterium, Lactobacillus*) and reducing pro-inflammatory species, thereby shaping a more favorable BAs profile (Shen et al., 2017; Di Meo et al., 2019). The well-documented anti-inflammatory, antioxidant, and neuroprotective effects of curcumin in models of depression and AD could be intimately intertwined with its ability to modulate BAs signaling and the gut-brain axis. Curcumin has been shown to ameliorate depressive-like behaviors in rodents, effects associated with normalized HPA axis activity and increased BDNF levels, which may be facilitated by its impact on the gut-BA-brain circuit (Zhang et al., 2012; Lopresti, 2017).

**Berberine (an isoquinoline alkaloid from plants like** ***Coptis chinensis*** **and** ***Berberis*** ***vulgaris*****):** Berberine is a potent gut microbiome modulator with a unique mechanism. It exerts a primary indirect effect by inhibiting bacterial BSH activity, leading to an accumulation of conjugated BAs in the gut lumen (Gu et al., 2015; Wang et al., 2022a). This accumulation of conjugated BAs can enhance FXR signaling specifically in the intestine, leading to a robust increase in FGF15/19 production. FGF15/19 then travels to the liver to repress *CYP7A1* and hepatic BA synthesis, resulting in improved glucose homeostasis and reduced lipogenesis (Guo et al., 2016; Pathak et al., 2018). The resulting systemic anti-inflammatory and metabolic improvements resonate in the brain. Berberine has demonstrated efficacy in animal models of depression and AD, improving cognitive function, reducing depressive-like behaviors, and attenuating neuroinflammation, effects that are likely mediated by this microbiome-BA-FXR/TRG5 axis (Zhu et al., 2011).

**Quercetin (a flavonoid abundant in onions, apples, and capers):** This flavonoid can alter BAs composition by modulating the gut bacterial community and has been identified as a TGR5 activator, promoting GLP-1 secretion from intestinal L-cells (Zhang et al., 2016; Les et al., 2018). Its potent anti-inflammatory and antioxidant properties, combined with its ability to modulate BAs signaling and the gut-brain axis, contribute to its observed neuroprotective benefits in models of AD, cerebral ischemia, and aging-related cognitive decline (Costa et al., 2016; Dajas et al., 2003).

### 5.2 Bioactive compounds in tea

Tea, one of the most widely consumed beverages globally, is not only a cultural symbol but also a treasure trove of diverse bioactive compounds. Its primary active constituents include catechins (particularly epigallocatechin gallate, EGCG), theanine, caffeine, theaflavins, and tea polysaccharides. The various bioactive compounds in tea constitute a complex “multi-component, multi-target” regulatory network that collectively acts on multiple aspects of BAs synthesis, metabolism, signaling, and gut microecology. By modulating FXR/TGR5 signaling, reshaping the gut microbiota to optimize the BAs pool composition, and exerting direct neuroprotective and anti-inflammatory effects, these compounds hold promise for positively influencing the prevention and adjunctive treatment of neuropsychiatric disorders such as Alzheimer's disease, Parkinson's disease, and depression (Xu et al., 2023; Tang et al., 2019; Yan et al., 2020).

#### 5.2.1 Modulation of bile acid receptors and synthases by tea polyphenols

Tea polyphenols, especially EGCG, are the most extensively studied active substances in tea. Researches show that EGCG can function as a modulator of the FXR. Although its direct binding affinity for FXR is relatively weak, EGCG can indirectly regulate FXR transcriptional activity by influencing FXR co-activators or through other upstream signaling pathways, such as the MAPK pathway (Li and Chiang, 2012; Xu et al., 2016). In animal models, EGCG intervention has been shown to downregulate the expression of the rate-limiting BAs synthesis enzyme CYP7A1 in the liver, thereby reducing total BAs synthesis and altering the BAs composition profile—for instance, decreasing the proportion of DCA (Huang et al., 2018). Furthermore, EGCG is an effective agonist of Takeda G protein-coupled receptor 5 (TGR5). By activating TGR5, EGCG promotes the secretion of glucagon-like peptide-1 (GLP-1) from intestinal L-cells. This not only aids in improving glucose homeostasis but also contributes to brain health through the neuroprotective and anti-inflammatory effects of GLP-1 (Cremonini et al., 2021). The anti-inflammatory signaling mediated by TGR5 synergizes with the inherent antioxidant and anti-inflammatory properties of EGCG. Together, they collectively maintain the integrity of BBB and neuronal function (Mokra et al., 2022).

#### 5.2.2 Reshaping the gut microbiota and secondary BAs metabolism by tea components

Tea components significantly shape the gut microbial ecosystem. EGCG and theaflavins possess antimicrobial activity, selectively inhibiting the growth of potential pathogens (e.g., certain Clostridium species) while promoting the proliferation of beneficial bacteria such as Bifidobacterium and Lactobacillus (Patra, 2024). This alteration in microbial community structure directly impacts secondary BAs production. Specifically, EGCG can inhibit bacterial bile salt hydrolase (BSH) activity, leading to an increased proportion of conjugated BAs in the gut, which may potentiate intestinal FXR signaling. Concurrently, it can suppress 7α-dehydroxylase activity, reducing the conversion of CA to DCA and thereby lowering the concentration of potentially cytotoxic secondary BAs like DCA in serum and feces (Wen et al., 2023). This ability to shift the bile acid pool toward a more hydrophilic and less toxic profile is a key mechanism through which tea components alleviate systemic inflammation and protect hepatic and neurological function.

#### 5.2.3 Synergistic and balancing effects of theanine and caffeine

Theanine is a unique non-protein amino acid found almost exclusively in tea, known for its notable neuroregulatory and relaxant effects. It can cross BBB, influencing neurotransmitter levels in the brain—such as increasing GABA, dopamine, and serotonin—while antagonizing the excitotoxic effects of glutamate (Lardner, 2014). In the context of BAs metabolism, theanine exerts anti-stress and hypothalamic-pituitary-adrenal (HPA) axis modulating effects, which may indirectly stabilize the enterohepatic circulation of BAs against disruptions caused by psychological stress (Rothenberg and Zhang, 2019). Caffeine, a central nervous system stimulant, has less direct research linking it to BAs metabolism, but it is known to exert neuroprotective effects by antagonizing adenosine A2A receptors. Importantly, in tea, theanine effectively mitigates the potential tension and anxiety sometimes induced by caffeine. This unique “slow-release” and “balancing” effect makes tea a gentle and sustainable neuromodulator, potentially providing an ideal medium for maintaining a stable bile acid-gut-brain axis function over the long term (Unno et al., 2011).

#### 5.2.4 Prebiotic-like effects of tea polysaccharides

Tea polysaccharides are a class of complex macromolecules in tea that are not easily absorbed directly by the human digestive tract but can serve as fermentation substrates for gut microbiota, exerting prebiotic-like effects. Studies have found that tea polysaccharides significantly increase the abundance of short-chain fatty acid (SCFA)-producing bacteria, such as certain genera within the Bacteroidetes and Firmicutes phyla (Wang et al., 2022b). The resultant increase in SCFA production (particularly butyrate) lowers colonic pH, further inhibiting the activity of 7α-dehydroxylating bacteria, thereby creating an intestinal environment less conducive to the generation of secondary bile acids like lithocholic acid (LCA) and DCA. Furthermore, SCFAs themselves possess potent anti-inflammatory and immunomodulatory functions and can enhance the intestinal barrier, preventing systemic inflammation triggered by endotoxin translocation (Yang et al., 2025). Through this pathway, tea polysaccharides indirectly but powerfully optimize the intestinal microenvironment for bile acid metabolism, laying a solid foundation for upstream bile acid signaling regulation (Chen et al., 2024).

### 5.3 Dietary fibers

**Soluble Fiber (e.g., inulin, pectin, found in oats, legumes, and fruits):** This represents an indirect, prebiotic mechanism. Fermentable fibers are not digested in the small intestine and serve as substrates for colonic bacteria. Their fermentation produces Short-Chain Fatty Acids (SCFAs) like acetate, propionate, and butyrate (Cani et al., 2009). SCFAs, particularly butyrate, lower the intestinal pH, creating an environment that inhibits the growth and activity of 7α-dehydroxylating bacteria (e.g., *Clostridium scindens*), thereby reducing the production of secondary BAs like DCA and LCA (Ridlon et al., 2016b; Berding et al., 2021). A shift toward a less hydrophobic BAs pool, with relatively higher levels of primary BAs, may be beneficial for reducing systemic and neuro-inflammation. Epidemiological studies consistently link diets high in soluble fiber with improved cognitive function and a reduced risk of AD and vascular dementia, potentially mediated in part through this BA-modulating mechanism (van de Wouw et al., 2018).

### 5.4 Probiotics

Probiotics can regulate BAs metabolism through various mechanisms. Probiotics such as *Lactobacillus* and *Bifidobacterium* can express BAs hydrolases, which affect the process of BAs dissociation (Begley et al., 2006). At the same time, probiotics can also reduce the number of 7α-dehydrogenating bacteria and lower the production of secondary BAs through competitive inhibition (O'Toole and Cooney, 2008). Probiotics such as oligofructose and oligogalactose can selectively promote beneficial bacterial growth and indirectly regulate BAs metabolism (Roberfroid et al., 2010). Research has shown that prebiotic intervention can alter the structure of gut microbiota, optimize BAs composition, and improve metabolic health (Gibson et al., 2017). Synbiotics (a combination of probiotics and prebiotics) may have a synergistic effect in regulating BAs metabolism. Clinical studies have shown that specific synbiotics can significantly alter BAs profiles, improve insulin sensitivity, and metabolic parameters (Zhang et al., 2021). These findings provide new ideas for regulating BAs metabolism through microbial intervention.

### 5.5 Resistant starch (found in cooked and cooled potatoes, green bananas, legumes)

Similar to other prebiotics, resistant starch promotes the growth of specific beneficial bacteria, such as *Bifidobacterium, Lactobacillus*, and *Ruminococcus bromii*, which generally lack 7α-dehydroxylase activity (Martinez et al., 2010; Upadhyaya et al., 2016). This selective enrichment shapes a BA profile characterized by higher levels of primary BAs and lower levels of cytotoxic secondary BAs like LCA. This modulation has been mechanistically linked to improved glycemic control, enhanced gut barrier integrity, and reduced systemic inflammation—all factors that are beneficial for brain health and cognitive function (Cani et al., 2009; Bindels et al., 2015).

### 5.6 Taurine and glycine

Since the hepatic conjugation process is a major determinant of BAs physicochemical and signaling properties, dietary intake of the precursor amino acids taurine (abundant in seafood and meat) and glycine can influence the conjugation pattern of the BAs pool (Hofmann and Hagey, 2008). Taurine-conjugated BAs, in particular, have been specifically associated with the neuroprotective effects of TUDCA. Ensuring adequate dietary taurine may therefore be beneficial to the endogenous production of taurine-conjugated BAs, potentially enhancing the brain's resilience to stress and injury (Boatright et al., 2009; Ripps and Shen, 2012). Taurine supplementation itself has shown independent neuroprotective effects in various models of stroke, epilepsy, and neurodegeneration, which may be partly attributed to its role in supporting the conjugation and thus the function of the BAs pool (Ripps and Shen, 2012; Menzie et al., 2014).

## 6 Conclusions and perspectives

This comprehensive review has charted the remarkable scientific journey of BAs, from their classical role as simple digestive detergents to their contemporary status as central regulators of systemic metabolism and neurological health. We have outlined their complex, multi-pathway biosynthesis in the liver and their intricate enterohepatic circulation, emphasizing the indispensable role of the gut microbiome as a “bio-transformer” that generates a diverse BAs metabolome with distinct signaling properties. The activation of FXR and TGR5 receptors by specific BAs forms a pervasive signaling network. This network extends far beyond the liver and gut. It directly and indirectly influences key brain processes, including neuroinflammation, cellular stress, neurotransmitter function, synaptic plasticity, and BBB integrity. The expression, regulation, and relative importance of FXR and TGR5 signaling within the CNS vs. via peripheral pathways (e.g., vagal, immune) need further clarification.

A growing, though largely correlative, body of evidence associates dysregulation of the BA signaling system with a spectrum of brain disorders. From Alzheimer's and Parkinson's diseases to major depression and hepatic encephalopathy, a common observation is the alteration of BAs profiles—frequently manifesting as a reduction in beneficial secondary BAs and a concomitant increase in toxic hydrophobic species. This recurrent “dysbiotic” signature points to a core disruption of the gut-liver-brain axis across diverse neuropathologies, suggesting that BAs metabolism may represent a convergent pathway linking peripheral metabolic and inflammatory states to brain health.

The emerging field of nutritional neuroscience offers a compelling and pragmatic strategy which is the targeted modulation of the BAs system through diet. As detailed, specific phytochemicals like resveratrol, curcumin, and berberine, along with prebiotic fibers and specific amino acids, demonstrate a significant capacity to reshape the BAs pool and fine-tune receptor signaling. These dietary components act as natural “BAs modulators,” presenting a viable, non-pharmacological path for prevention and adjunct therapy by harnessing the body's own endogenous signaling systems to promote a neuroprotective environment. However, several challenges and exciting frontiers must be addressed to translate this knowledge into effective clinical applications.

**Establishing Causality:** While strong associations between BAs alterations and neurological diseases are evident, more sophisticated research is still needed to establish definitive causality. The use of germ-free animals, targeted bacterial interventions (e.g., precise probiotic cocktails or fecal microbiota transplantation from characterized donors), and BAs receptor knockout models will be crucial to determine whether BA changes are primary drivers of neuropathology or secondary consequences of the disease state.

**BBB Dynamics and Brain Delivery:** A deeper, more nuanced understanding of how different BAs traverse the BBB under healthy and diseased conditions is essential. The expression, regulation, and function of specific BAs transporters (e.g., ASBT, OSTα/OSTβ, MRPs) at the BBB remain an active area of investigation. Developing strategies to enhance the brain delivery of neuroprotective BAs or BAs-based drugs, perhaps through nano-formulations or prodrug approaches, could significantly improve their therapeutic efficacy.

**Personalized Nutrition and Medicine:** The effects of any dietary intervention on BAs metabolism are highly dependent on an individual's baseline gut microbiota composition, their genetic background (e.g., polymorphisms in BA receptors and transporters), and their overall metabolic health. Future therapeutic strategies will likely need to be highly personalized. This entails deep phenotyping through microbiome sequencing, BAs metabolomic profiling, and host genetics.

**Robust Clinical Translation:** While preclinical data, particularly for compounds like TUDCA, are exceptionally strong and promising, there is a pressing need for larger, well-designed, randomized, placebo-controlled human clinical trials to confirm their efficacy and safety in specific neurological diseases. Defining optimal dosing regimens, treatment durations, and identifying the patient subpopulations most likely to benefit are critical next steps.

**Novel Therapeutic Agonists/Antagonists:** The pharmaceutical development of highly specific, brain-penetrant FXR and TGR5 agonists, antagonists, or modulators that can target neurological pathways with minimal peripheral side effects is a highly active and promising area of research. Such compounds could offer more precise control over BA signaling than broad dietary interventions.

**Microbiome Engineering:** Beyond diet, more direct strategies aimed at manipulating the gut microbiome to correct BA dysregulation represent a futuristic but plausible frontier. This could include next-generation probiotics (e.g., engineered consortia containing *Clostridium scindens* to boost secondary BA production) or defined microbial ecosystem therapeutics tailored to restore a healthy BAs-metabolizing community.

In conclusion, the BAs system represents a fundamental biological pathway that intricately links our diet, our gut microbial symbionts, and our brain health. Harnessing this system through the sophisticated application of nutritional science holds immense promise for tackling the growing global burden of neurological and psychiatric diseases in the twenty-first century. It is critical to note that these promising results are primarily derived from cell and rodent models. Translating these findings to human pathophysiology requires caution due to significant species differences in BAs composition, receptor biology, and disease etiology. A deeper understanding of “bile-acid-mediated nutrition” could pave the way for a new class of evidence-based nutraceuticals and refined dietary guidelines specifically designed to optimize brain function and resilience across the human lifespan.
